# Cybersight: improving remote access to surgical training and mentoring

**Published:** 2023-12-01

**Authors:** Maria Jose Montero, Hannah Marr, Nathan Congdon, Meryem Altun, Alana Calise

**Affiliations:** 1 Associate Director: Clinical Services, FEH, Orbis, Puebla, Mexico.; 2 Pre-med student: New York University, New York, USA.; 3 Ulverscroft Chair of Global Eye Health: Queens University Belfast & Orbis International, Royal Victoria Hospital, Belfast, Ireland, UK.; 4 Program Manager: Orbis, Montreal, Canada.; 5 Program Manager: Orbis, Massachusetts, USA.

**Remote mentoring can provide affordable access to surgical training, even in low-resource settings.**

**Figure:**
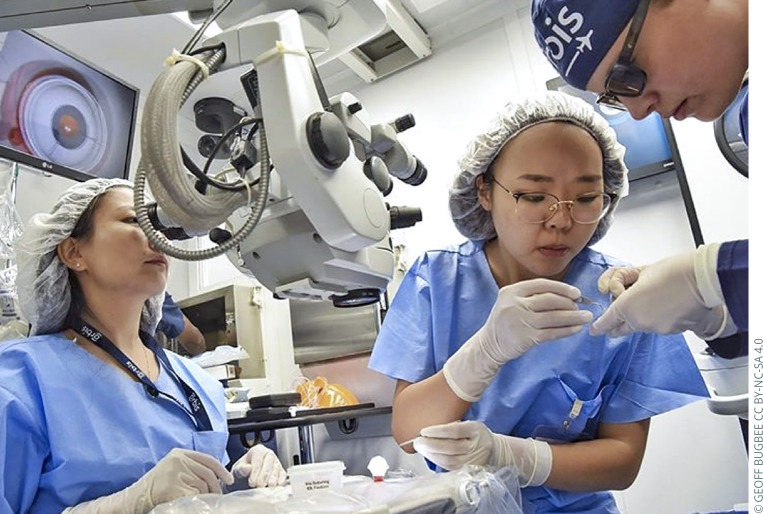
Residents undergoing anterior segment simulation training onboard the Orbis Flying Eye Hospital, a form of training that was hampered by the COVID-19 pandemic. **MONGOLIA**

Cataract remains the leading cause of blindness, disproportionately affecting low- and middle-income countries.^1^ As global populations continue to age, cataract-related vision impairment is projected to rise. A survey conducted in 2017 across twenty low- and middle-income countries revealed that only 36.7% of operable cataracts had been successfully treated.^2^ To address this pressing issue, an increase in trained ophthalmologists is required to provide high-quality, accessible surgery.^3^

Regrettably, some ophthalmology residency programmes suffer a shortage of mentored, hands-on surgical training opportunities, while others offer no surgical training at all.^4^ Recognising this disparity, Orbis has been making significant efforts, since 1982, to bring teaching physicians together with those in need of mentorship. However, the COVID-19 pandemic profoundly impacted hands-on surgical training models, such as hospital-based training, the Flying Eye Hospital, and face-to-face fellowships.

To continue supporting eye health professionals worldwide, Orbis has developed remote surgical mentorship models.

Remote mentoring in surgical skills is a method of professional development whereby an expert ophthalmologist uses live video and audio feedback to guide a less experienced ophthalmologist (or ophthalmology resident) during live or simulated surgery, regardless of their geographical distance from one another. Remote surgical mentorship offers unique advantages, including increased access to hands-on training and exposure to a diverse range of mentors using a range of different approaches. This can be challenging to achieve through traditional in-person mentoring.

Simulation training improves the hand-eye coordination of trainee surgeons before they transition into an actual operating room setting. It also has the potential to improve patient safety and enhance outcomes while also maximising the impact of scarce teaching resources. Improving trainees’ access to simulation training is therefore critical for helping them to develop their skills in a safe environment, thereby reducing patient complications during their training.

It is possible to pay up to $20,000 USD for live surgical mentorship equipment and systems. In our experience, however, it is possible to do this for much less. For example, a simple operating microscope can be used with artificial eyes, while streaming the learner's feed over Zoom (www.zoom.com) using the free version of the Microrec app (https://customsurgical.co/microrec-app/). The learners join the video call via their phones or tablets and can then engage directly with the Orbis Volunteer Faculty mentors via audio and video. There are sometimes bandwidth or connectivity issues, depending on the location and local internet infrastructure. However, the bandwidth needed for access to platforms such as Zoom is generally sufficient.

Within Orbis programmes, remote surgical mentorship usually develops out of existing long-term relationships with clinical partners in low-or middle-income countries (see case studies) and are not currently offered to the general public. However, anyone can create an account on Orbis’ digital training and telehealth platform, Cybersight.org, and immediately gain access, at no cost, to the same online courses used as part of the remote training. Access to free courses and webinars are also available to everyone with a Cybersight account. An additional feature, e-Consultation, requires users to have an active medical license in one of the low-resource countries included in this list: https://cybersight.org/where-we-work.

## Case study 1. Remote, asynchronous wet lab training in manual small incision cataract surgery

One of our first surgical mentoring projects, back in 2020, involved instructing ophthalmology residents in India in manual small-incision cataract surgery (MSICS).

**Figure:**
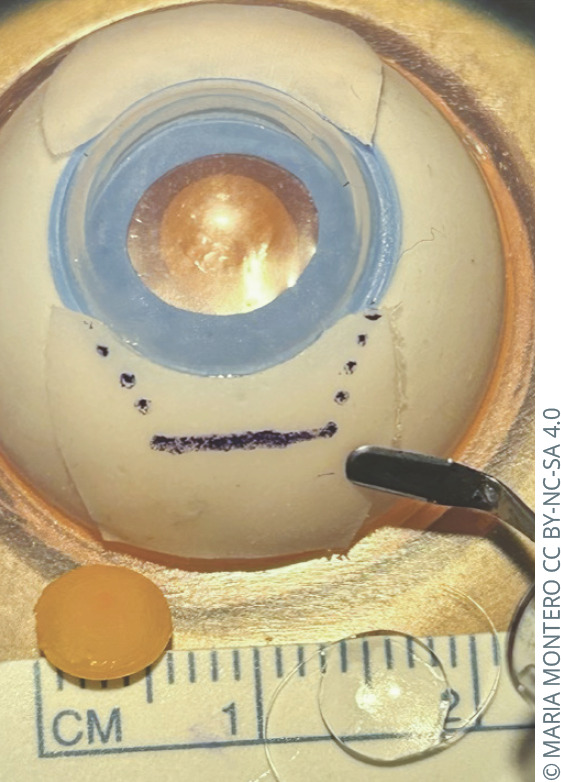
MICS plastic eye model used for simulation training.

Trainees signed into the Cybersight platform and attended one live lecture a week, covering one of the specific steps in MSICS. Residents recorded two videos in the wet lab, practicing the surgical step of the week on artificial eyes – one before attending the live lecture, and one after attending it. These pre- and post-lecture surgical simulation videos were uploaded to the Cybersight platform, where they were masked, anonymised and then graded using the Ophthalmic Simulated Surgical Competency Assessment Rubric for MSICS.^1^ The residents also completed an anonymous post-training satisfaction survey.

Nine residents successfully completed the training, submitting a total of 54 surgical simulation videos. The residents’ average competency score increased by 5.6 points on average, which was a statistically significant improvement. Post-training satisfaction survey results indicated improved knowledge (average score of 8.7 out of 10), satisfaction with the course (8.6 out of 10), and a willingness to recommend this course to other eye health professionals (8.7 out of 10).

This virtual wet lab training yielded a substantial enhancement in the surgical simulation skills of participating residents. Such training models can be successfully applied in locations where access to in-person training is challenging and can also support residency curriculums.

**Figure:**
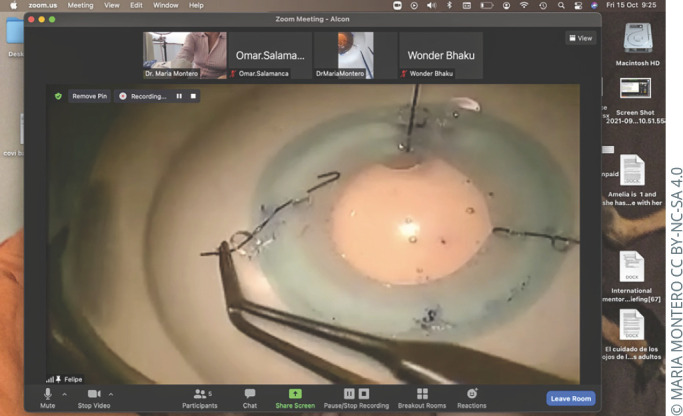
Remote training in advanced cataract skills at a wet lab in Chile.

## Case study 2. Remote, synchronous wet lab training in advanced phacoemulsification skills

A more recent model of remote wet lab training focused on improving advanced phacoemulsification skills. The goal was to train six learners via synchronous (real-time) online simulation. For four months, learners joined two-day online simulation workshops, at two locations in Santiago, Chile. Learners connected their simulation microscope to an online video call and practiced their advanced phacoemulsification techniques on artificial eyes in real time, while Orbis Volunteer Faculty mentors located in either Mexico or the United States provided guidance and feedback. On day two of the monthly workshop, learners and mentors convened for follow-up lectures and discussion of the hands-on simulation training sessions of the previous day. Based on learners’ experience levels, the virtual simulation training included four topics: anterior vitrectomy, iris suturing, aphakia, and small pupil. Participants’ survey results showed increasing levels of comfort as the workshops progressed.

**Figure:**
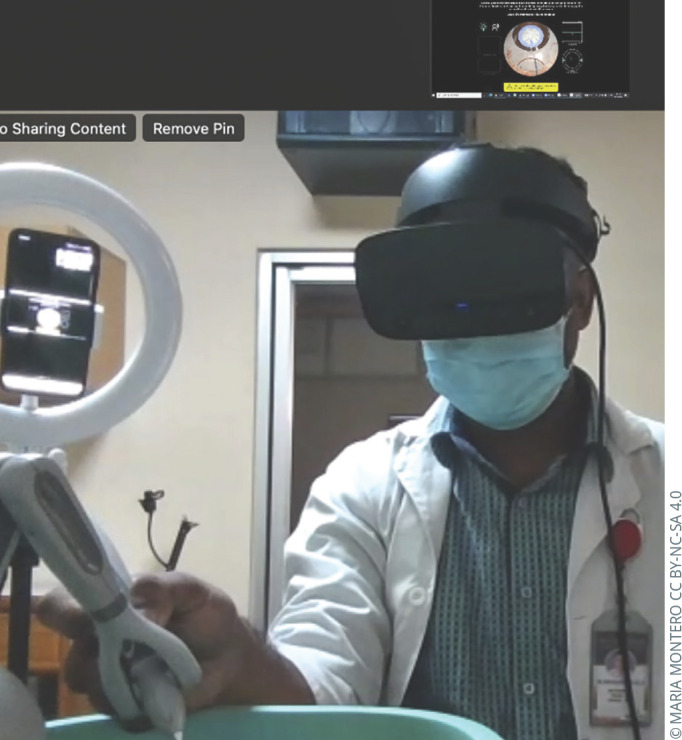
Surgeons using the VR simulator can be observed remotely via a live video call.

## Case study 3. Remote, synchronous virtual reality training

Orbis is working on a novel virtual reality (VR) MSICS simulation system in partnership with FundamentalVR (https://fundamentalsurgery.com) to explore the role of VR technology in remote surgical training.

The aim is to create a VR surgery experience that surgeons can share with their mentors via remote video call, in real time. The simulator will ultimately be able to score active participants on the quality of their technique, giving quantitative feedback on mistakes, while a mentor provides guidance and qualitative feedback at the end of the session.

A prototype system was implemented at six partner institutions in Bangladesh, Mongolia, India, and Ethiopia, where residents took part in VR training focused on improving MSICS skills over the course of one 40-hour week. Participant surveys indicated an increase in confidence in MSICS surgical skills at the end of the week.

Development is ongoing and Orbis is looking to launch the system in 2024. At this time, the technology is too new to estimate an exact price and to describe details around accessibility, but this will become clear as the platform continues to develop.

## References

[1] Flaxman SR Bourne RRA Resnikoff S et al. Global causes of blindness and distance vision impairment 1990–2020: a systematic review and meta-analysis. Lancet Glob H. 2017;5(12):e1221-e1234. 10.1016/S2214-109X(17)30393-5 29032195

[2] Ramke J Gilbert CE Lee AC Ackland P Limburg H Foster A. Effective Cataract Surgical Coverage: An Indicator for Measuring Quality-of-Care in the Context of Universal Health Coverage. PLoS ONE. 2017;12(3):e0172342. 28249047 10.1371/journal.pone.0172342 PMC5382971

[3] Resnikoff S Felch W Gauthier TM Spivey B. The number of ophthalmologists in practice and training worldwide: A growing gap despite more than 200,000 practitioners. Br J Ophthalmol. 2012;96(6):783. 22452836 10.1136/bjophthalmol-2011-301378

[4] Thomas R Dogra M. An evaluation of medical college departments of ophthalmology in India and change following the provision of modern instrumentation and training. Indian J Ophthalmol. 2008;56(1):9-16. 18158398 10.4103/0301-4738.37589 PMC2636055

[5] Geary A Wen Q Adrianzén R, et al. The impact of distance cataract surgical wet laboratory training on cataract surgical competency of ophthalmology residents. BMC Med Educ 2021;21:219. 33874941 10.1186/s12909-021-02659-y PMC8054504

[6] Dean WH Murray NL Buchan JC Golnik K Kim MJ Burton MJ. Ophthalmic Simulated Surgical Competency Assessment Rubric for manual small-incision cataract surgery. J Cataract Refract Surg. 2019;45(9):1252. 31470940 10.1016/j.jcrs.2019.04.010 PMC6727782

